# Illinois Veterinary Medical Association

**Published:** 1895-05

**Authors:** Albert Babb

**Affiliations:** Secretary


Secretary.

### ILLINOIS STATE VETERINARY MEDICAL ASSOCIATION.

The meeting of the Illinois State Veterinary Medical Association was held at Peoria, Ill., February 28th, 1895. The meeting was called to order at 11 A.M. by the President, Dr. M. Wilson, of Mendota, Ill.

On roll-call by the Secretary the following members responded: Drs. A. G. Alverson, G. Z. Barnes, A. Babb, Geo. Ditewig, C. D. Hartman, R. W. Story, N. I. Stringer, A. M. Story, and M. Wilson.

On motion, Dr. W. C. Hanawalt, a graduate of the Chicago Veterinary College, class of 1892, who was vouched for by Dr. A. M. Story, was elected to membership by acclamation. The Society then adjourned for dinner. On convening at 2 P.M., the Society listened to a paper by Dr. Ditewig entitled "The Strongyli Tetracanthus and Armatus as a Cause of Death." A lengthy discussion then took place in which most of the members present related their experience with these helminths. On call of the President Dr. Barnes next read his essay "What of the Future of the Veterinary Profession?"

This article called for a general discussion, after which Dr. Babb gave a paper on "The Etiology of Tuberculosis."

At the close of the discussion that followed, Dr. Stringer made a motion, which was seconded by Dr. Ditewig, that the President extend in our names the regrets of the Society to Dr. J. W. Parkinson in his present illness and express our hopes that he may recover. On motion the Society adjourned to meet in Chicago next November.

ALBERT BABB, A.B., M.D.C.,
Secretary.

